# *S*-Phenyl benzothio­ate

**DOI:** 10.1107/S1600536812037142

**Published:** 2012-09-01

**Authors:** Yonas H. Belay, Henok H. Kinfe, Alfred Muller

**Affiliations:** a Research Centre for Synthesis and Catalysis, Department of Chemistry, University of Johannesburg (APK Campus), PO Box 524, Auckland Park, Johannesburg, 2006, South Africa

## Abstract

In the title compound, C_13_H_10_OS, the phenyl rings are inclined to one another by 51.12 (8)°. There is a short C—H⋯S contact in the molecule.In the crystal, molecules are linked *via* C—H⋯O hydrogen bonds forming chains along the *a* axis. Molecules are also linked by C—H⋯π and weak π–π interactions [centroid–centroid distance = 3.9543 (10) Å].

## Related literature

The title compound was obtained by the reaction of thiophenolyate and benzoyl chloride in an alkaline medium. For background to the title compound, see: Reddy *et al.* (2010 ▶); Katritzky *et al.* (2007 ▶). For details of the Cambridge Structural Database, see: Allen (2002 ▶).
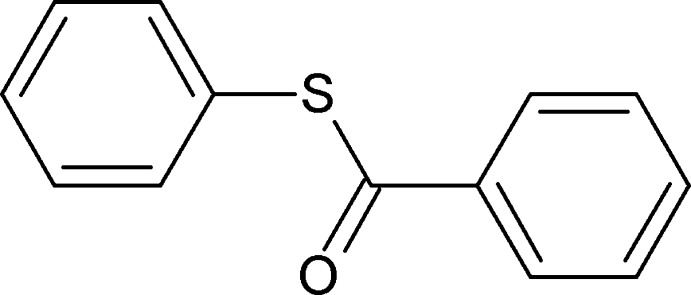


## Experimental

### 

#### Crystal data

C_13_H_10_OS
*M* *_r_* = 214.27
Monoclinic, 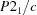

*a* = 5.7203 (1) Å
*b* = 15.1315 (3) Å
*c* = 12.0606 (3) Å
β = 96.867 (1)°
*V* = 1036.44 (4) Å^3^
*Z* = 4
Cu *K*α radiation
μ = 2.49 mm^−1^
*T* = 100 K
0.25 × 0.12 × 0.12 mm

#### Data collection

Bruker APEX DUO 4K-CCD diffractometer
Absorption correction: multi-scan (*SADABS*; Bruker, 2008 ▶) *T*_min_ = 0.575, *T*_max_ = 0.754
9164 measured reflections
1759 independent reflections
1702 reflections with *I* > 2σ(*I*)
*R*_int_ = 0.022

#### Refinement

*R*[*F* ^2^ > 2σ(*F* ^2^)] = 0.032
*wR*(*F* ^2^) = 0.082
*S* = 1.04
1759 reflections
136 parameters
H-atom parameters constrained
Δρ_max_ = 0.35 e Å^−3^
Δρ_min_ = −0.26 e Å^−3^

### 

Data collection: *APEX2* (Bruker, 2011 ▶); cell refinement: *SAINT* (Bruker, 2008 ▶); data reduction: *SAINT* and *XPREP* (Bruker, 2008 ▶); program(s) used to solve structure: *SIR97* (Altomare *et al.*, 1999 ▶); program(s) used to refine structure: *SHELXL97* (Sheldrick, 2008 ▶); molecular graphics: *DIAMOND* (Brandenburg & Putz, 2005 ▶); software used to prepare material for publication: *WinGX* (Farrugia, 1999 ▶).

## Supplementary Material

Crystal structure: contains datablock(s) global, I. DOI: 10.1107/S1600536812037142/ds2217sup1.cif

Structure factors: contains datablock(s) I. DOI: 10.1107/S1600536812037142/ds2217Isup2.hkl

Supplementary material file. DOI: 10.1107/S1600536812037142/ds2217Isup3.cml

Additional supplementary materials:  crystallographic information; 3D view; checkCIF report

## Figures and Tables

**Table 1:** Hydrogen-bond geometry (Å, °) *Cg*1 and *Cg*2 are the centroids of the C2–C7 and C8–C13 rings, respectively.

*D*—H⋯*A*	*D*—H	H⋯*A*	*D*⋯*A*	*D*—H⋯*A*
C7—H7⋯S1	0.95	2.52	2.9592 (16)	109
C13—H13⋯O1^i^	0.95	2.56	3.4889 (18)	167
C10—H10⋯*Cg*1^ii^	0.95	2.97	3.506 (2)	117
C5—H5⋯*Cg*2^iii^	0.95	2.73	3.5915 (19)	152

Symmetry codes: (i) 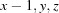; (ii) 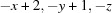; (iii) 
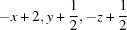
.

**Fig. 1.**
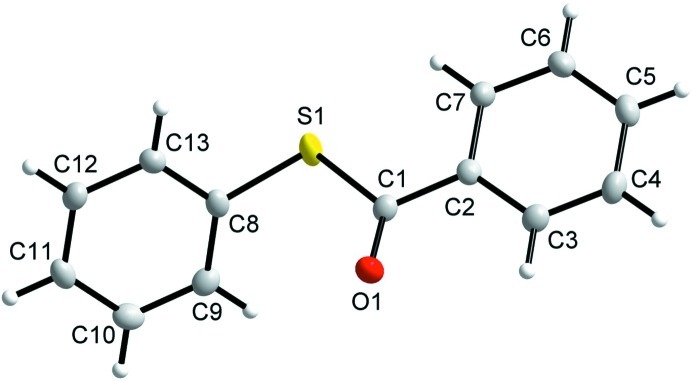
A view of (1). Displacement ellipsoids are drawn at the 50% probability level.

**Fig. 2.**
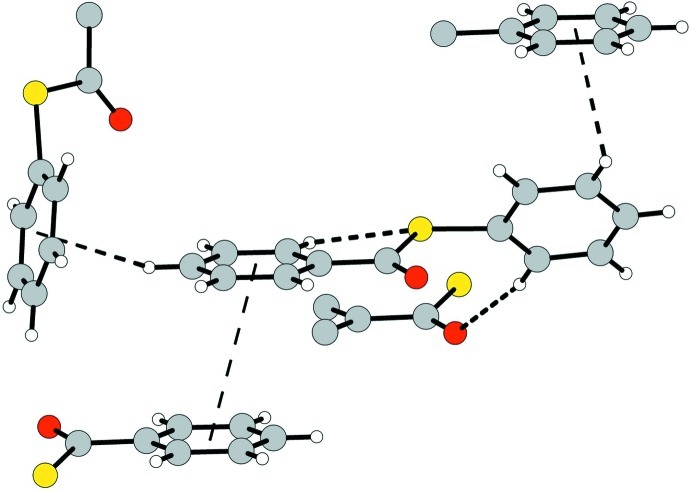
Packing diagram of (1) showing the C—H···O/S/π interactions as well as the π-π stacking.

## supplementary crystallographic information

### Comment

Reaction of thiophenolyate and benzyol chloride in alkaline medium was described previously by Reddy *et al.*, 2010. We have repeated the preparation of this compound to be used as starting material in some of our research. Benzoylation of thiophenol afforded colorless crystals of the title compound (see scheme and Figure 1) suitable for single crystal X-ray analysis of which the structure is reported herein. Molecules of the title compound crystalizes in the *P*2_1_/*c* (*Z*=4) space group. All bond lengths are within their normal ranges (Allen, 2002). In the crystal packing several C—H···O/S/π interactions (see table 1, Fig. 2) as well as π-π stacking are observed (centroid to centroid distance = 3.9543 (10) Å, ring slippage = 1.366 Å).

### Experimental

A mixture of sodium hydroxide (344 mg, 8.61 mmol) and thiophenol (0.9 ml, 8.61 mmol) were dissolved in methanol (22 ml) for about 10 minutes. Benzoyl chloride (1 ml) was added to it. The reaction mixture was stirred overnight and then poured into ice-cold water. Afterwards it was filtered and dried to afford the title compound as white crystals in 63% yield.

### Refinement

All hydrogen atoms were positioned in geometrically idealized positions with C—H = 0.95 Å and were allowed to ride on their parent atoms with *U*_iso_(H) = 1.2*U*_eq_. A discrepant reflection (1 3 2) was removed in the final stages of refinement

### Crystal data

**Table:**

C_13_H_10_OS	*F*(000) = 448
*M**_r_* = 214.27	*D*_x_ = 1.373 Mg m^−^^3^
Monoclinic, *P*2_1_/*c*	Cu *K*α radiation, λ = 1.54178 Å
Hall symbol: -P 2ybc	Cell parameters from 6683 reflections
*a* = 5.7203 (1) Å	θ = 4.7–65.8°
*b* = 15.1315 (3) Å	µ = 2.49 mm^−^^1^
*c* = 12.0606 (3) Å	*T* = 100 K
β = 96.867 (1)°	Rectangular, colourless
*V* = 1036.44 (4) Å^3^	0.25 × 0.12 × 0.12 mm
*Z* = 4	

### Data collection

**Table:**

Bruker APEX DUO 4K-CCD diffractometer	1759 independent reflections
Radiation source: Incoatec IµS microfocus X-ray source	1702 reflections with *I* > 2σ(*I*)
Incoatec Quazar Multilayer Mirror monochromator	*R*_int_ = 0.022
Detector resolution: 8.4 pixels mm^-1^	θ_max_ = 66.4°, θ_min_ = 4.7°
φ and ω scans	*h* = −2→6
Absorption correction: multi-scan (*SADABS*; Bruker, 2008)	*k* = −17→17
*T*_min_ = 0.575, *T*_max_ = 0.754	*l* = −14→13
9164 measured reflections	

### Refinement

**Table:**

Refinement on *F*^2^	Primary atom site location: structure-invariant direct methods
Least-squares matrix: full	Secondary atom site location: difference Fourier map
*R*[*F*^2^ > 2σ(*F*^2^)] = 0.032	Hydrogen site location: inferred from neighbouring sites
*wR*(*F*^2^) = 0.082	H-atom parameters constrained
*S* = 1.04	*w* = 1/[σ^2^(*F*_o_^2^) + (0.0364*P*)^2^ + 0.7558*P*] where *P* = (*F*_o_^2^ + 2*F*_c_^2^)/3
1759 reflections	(Δ/σ)_max_ = 0.001
136 parameters	Δρ_max_ = 0.35 e Å^−^^3^
0 restraints	Δρ_min_ = −0.26 e Å^−^^3^

### Special details

**Table:**

Experimental. The intensity data was collected on a Bruker Apex DUO 4 K CCD diffractometer using an exposure time of 5 s/frame. A total of 2274 frames were collected with a frame width of 1° covering up to θ = 66.38° with 96.3% completeness accomplished.Analytical data: mp: 53–55 °C (Lit. 54–55 °C; Katritzky *et al.*, 2007); ^1^H NMR (CDCl_3_, 400 MHz): d 8.03 (d, *J* = 0.8 Hz, 1H), 8.01(d, *J* = 1.2 Hz, 1H), 7.62–7.58 (m, 1H), 7.52–7.44 (m, 7H), ^13^C NMR (CDCl_3_, 400 MHz): d 190.1, 136.6, 135.1, 133.6, 129.5, 129.2, 128.7, 127.5, 127.3.
Geometry. All e.s.d.'s (except the e.s.d. in the dihedral angle between two l.s. planes) are estimated using the full covariance matrix. The cell e.s.d.'s are taken into account individually in the estimation of e.s.d.'s in distances, angles and torsion angles; correlations between e.s.d.'s in cell parameters are only used when they are defined by crystal symmetry. An approximate (isotropic) treatment of cell e.s.d.'s is used for estimating e.s.d.'s involving l.s. planes.
Refinement. Refinement of *F*^2^ against ALL reflections. The weighted *R*-factor *wR* and goodness of fit *S* are based on *F*^2^, conventional *R*-factors *R* are based on *F*, with *F* set to zero for negative *F*^2^. The threshold expression of *F*^2^ > σ(*F*^2^) is used only for calculating *R*-factors(gt) *etc*. and is not relevant to the choice of reflections for refinement. *R*-factors based on *F*^2^ are statistically about twice as large as those based on *F*, and *R*- factors based on ALL data will be even larger.

### Fractional atomic coordinates and isotropic or equivalent isotropic displacement parameters (Å^2^)

**Table:**

	*x*	*y*	*z*	*U*_iso_*/*U*_eq_	
S1	0.66521 (7)	0.44609 (2)	0.09450 (3)	0.02499 (16)	
O1	1.05856 (19)	0.36323 (7)	0.17500 (9)	0.0242 (3)	
C1	0.9304 (3)	0.42516 (10)	0.18525 (12)	0.0180 (3)	
C2	0.9751 (3)	0.49420 (10)	0.27395 (12)	0.0174 (3)	
C3	1.1950 (3)	0.49605 (10)	0.33797 (13)	0.0210 (3)	
H3	1.3106	0.4531	0.3262	0.025*	
C4	1.2443 (3)	0.56059 (11)	0.41871 (14)	0.0237 (4)	
H4	1.3943	0.5619	0.462	0.028*	
C5	1.0767 (3)	0.62332 (11)	0.43703 (13)	0.0234 (3)	
H5	1.1117	0.6673	0.4928	0.028*	
C6	0.8580 (3)	0.62174 (11)	0.37383 (13)	0.0239 (4)	
H6	0.7429	0.6647	0.3863	0.029*	
C7	0.8067 (3)	0.55781 (11)	0.29258 (13)	0.0214 (3)	
H7	0.6566	0.5571	0.2493	0.026*	
C8	0.6486 (3)	0.35922 (10)	−0.00504 (12)	0.0182 (3)	
C9	0.8130 (3)	0.35150 (10)	−0.08087 (13)	0.0211 (3)	
H9	0.9497	0.3877	−0.0737	0.025*	
C10	0.7749 (3)	0.29047 (11)	−0.16688 (13)	0.0231 (4)	
H10	0.8871	0.2843	−0.2184	0.028*	
C11	0.5735 (3)	0.23835 (10)	−0.17811 (13)	0.0221 (3)	
H11	0.5472	0.1972	−0.2378	0.027*	
C12	0.4107 (3)	0.24634 (10)	−0.10217 (13)	0.0210 (3)	
H12	0.2734	0.2105	−0.1097	0.025*	
C13	0.4483 (3)	0.30674 (10)	−0.01499 (12)	0.0192 (3)	
H13	0.3376	0.312	0.0374	0.023*	

### Atomic displacement parameters (Å^2^)

**Table:**

	*U*^11^	*U*^22^	*U*^33^	*U*^12^	*U*^13^	*U*^23^
S1	0.0287 (3)	0.0191 (2)	0.0243 (2)	0.00622 (15)	−0.00893 (16)	−0.00571 (15)
O1	0.0236 (6)	0.0242 (6)	0.0242 (6)	0.0056 (5)	0.0007 (4)	−0.0028 (5)
C1	0.0190 (7)	0.0164 (7)	0.0181 (8)	−0.0014 (6)	0.0004 (6)	0.0032 (6)
C2	0.0203 (7)	0.0174 (8)	0.0146 (7)	−0.0018 (6)	0.0027 (6)	0.0027 (6)
C3	0.0211 (8)	0.0189 (8)	0.0224 (8)	0.0007 (6)	0.0002 (6)	0.0032 (6)
C4	0.0235 (8)	0.0246 (8)	0.0215 (8)	−0.0034 (6)	−0.0043 (6)	0.0024 (6)
C5	0.0298 (8)	0.0226 (8)	0.0174 (7)	−0.0044 (6)	0.0015 (6)	−0.0019 (6)
C6	0.0245 (8)	0.0247 (8)	0.0233 (8)	0.0008 (6)	0.0055 (6)	−0.0040 (7)
C7	0.0188 (8)	0.0259 (9)	0.0191 (8)	0.0003 (6)	0.0010 (6)	−0.0019 (6)
C8	0.0223 (8)	0.0148 (7)	0.0161 (7)	0.0031 (6)	−0.0030 (6)	0.0010 (6)
C9	0.0203 (8)	0.0205 (8)	0.0220 (8)	−0.0013 (6)	0.0006 (6)	0.0060 (6)
C10	0.0248 (8)	0.0272 (9)	0.0181 (8)	0.0047 (6)	0.0054 (6)	0.0048 (6)
C11	0.0285 (8)	0.0196 (8)	0.0171 (8)	0.0045 (6)	−0.0021 (6)	−0.0021 (6)
C12	0.0195 (7)	0.0187 (8)	0.0242 (8)	−0.0010 (6)	−0.0005 (6)	−0.0006 (6)
C13	0.0202 (8)	0.0191 (8)	0.0184 (7)	0.0030 (6)	0.0024 (6)	0.0010 (6)

### Geometric parameters (Å, º)

**Table:**

S1—C8	1.7751 (15)	C6—H6	0.95
S1—C1	1.7894 (15)	C7—H7	0.95
O1—C1	1.2054 (19)	C8—C13	1.387 (2)
C1—C2	1.495 (2)	C8—C9	1.393 (2)
C2—C3	1.395 (2)	C9—C10	1.386 (2)
C2—C7	1.399 (2)	C9—H9	0.95
C3—C4	1.384 (2)	C10—C11	1.390 (2)
C3—H3	0.95	C10—H10	0.95
C4—C5	1.385 (2)	C11—C12	1.387 (2)
C4—H4	0.95	C11—H11	0.95
C5—C6	1.385 (2)	C12—C13	1.390 (2)
C5—H5	0.95	C12—H12	0.95
C6—C7	1.383 (2)	C13—H13	0.95
			
C8—S1—C1	104.81 (7)	C6—C7—H7	119.9
O1—C1—C2	124.23 (13)	C2—C7—H7	119.9
O1—C1—S1	123.81 (12)	C13—C8—C9	120.70 (14)
C2—C1—S1	111.96 (10)	C13—C8—S1	117.35 (12)
C3—C2—C7	119.34 (14)	C9—C8—S1	121.36 (12)
C3—C2—C1	118.40 (13)	C10—C9—C8	119.29 (14)
C7—C2—C1	122.24 (13)	C10—C9—H9	120.4
C4—C3—C2	119.85 (15)	C8—C9—H9	120.4
C4—C3—H3	120.1	C9—C10—C11	120.34 (14)
C2—C3—H3	120.1	C9—C10—H10	119.8
C3—C4—C5	120.60 (15)	C11—C10—H10	119.8
C3—C4—H4	119.7	C12—C11—C10	120.02 (15)
C5—C4—H4	119.7	C12—C11—H11	120
C6—C5—C4	119.80 (15)	C10—C11—H11	120
C6—C5—H5	120.1	C11—C12—C13	120.09 (14)
C4—C5—H5	120.1	C11—C12—H12	120
C7—C6—C5	120.21 (15)	C13—C12—H12	120
C7—C6—H6	119.9	C8—C13—C12	119.54 (14)
C5—C6—H6	119.9	C8—C13—H13	120.2
C6—C7—C2	120.20 (14)	C12—C13—H13	120.2
			
C8—S1—C1—O1	−0.46 (15)	C3—C2—C7—C6	−0.1 (2)
C8—S1—C1—C2	178.75 (10)	C1—C2—C7—C6	−178.42 (14)
O1—C1—C2—C3	10.8 (2)	C1—S1—C8—C13	122.78 (12)
S1—C1—C2—C3	−168.42 (11)	C1—S1—C8—C9	−66.00 (14)
O1—C1—C2—C7	−170.86 (15)	C13—C8—C9—C10	−0.1 (2)
S1—C1—C2—C7	9.93 (18)	S1—C8—C9—C10	−171.00 (11)
C7—C2—C3—C4	−0.1 (2)	C8—C9—C10—C11	0.8 (2)
C1—C2—C3—C4	178.26 (14)	C9—C10—C11—C12	−0.9 (2)
C2—C3—C4—C5	0.3 (2)	C10—C11—C12—C13	0.3 (2)
C3—C4—C5—C6	−0.2 (2)	C9—C8—C13—C12	−0.5 (2)
C4—C5—C6—C7	0.0 (2)	S1—C8—C13—C12	170.75 (11)
C5—C6—C7—C2	0.2 (2)	C11—C12—C13—C8	0.4 (2)

### Hydrogen-bond geometry (Å, º)

*Cg*1 and *Cg*2 are the centroids of the C2–C7 and C8–C13 rings, respectively.

**Table:**

*D*—H···*A*	*D*—H	H···*A*	*D*···*A*	*D*—H···*A*
C7—H7···S1	0.95	2.52	2.9592 (16)	109
C13—H13···O1^i^	0.95	2.56	3.4889 (18)	167
C10—H10···*Cg*1^ii^	0.95	2.97	3.506 (2)	117
C5—H5···*Cg*2^iii^	0.95	2.73	3.5915 (19)	152

Symmetry codes: (i) *x*−1, *y*, *z*; (ii) −*x*+2, −*y*+1, −*z*; (iii) −*x*+2, *y*+1/2, −*z*+1/2.
